# Occurrence of *Stenotrophomonas* carrying β-lactamase-encoding genes in a veterinary hospital environment

**DOI:** 10.1007/s11259-026-11539-z

**Published:** 2026-09-23

**Authors:** Maria Eduarda Rocha Jacques da Silva, Rafaela da Rosa Marques, Tainara Soares Weyh, Júlia Mendes Stropper, Laura Cadó Nemitz, Gabriela Merker Breyer, Lívia Kmetzsch, Silvia Dias de Oliveira, Franciele Maboni Siqueira

**Affiliations:** 1 Veterinary Bacteriology Laboratory, Faculty of Veterinary Medicine, Federal University of Rio Grande do Sul, Porto Alegre, 91540-000 Brazil; 2 Postgraduate Program in Veterinary Science, Federal University of Rio Grande do Sul, Porto Alegre, 91540-000 Brazil; 3 Laboratory of Molecular Biology of Pathogens, Center of Biotechnology, Federal University of Rio Grande do Sul, Porto Alegre, 91501-970 Brazil; 4 Laboratory of Immunology and Microbiology, School of Health and Life Sciences, Pontifical Catholic University of Rio Grande do Sul, Porto Alegre, 90619-900 Brazil

**Keywords:** Opportunistic pathogens, WGS, MDR, Carbapenem

## Abstract

*Stenotrophomonas* spp. are environmental bacteria increasingly recognized as opportunistic pathogens with multidrug resistance. This study characterized 17 environmental *Stenotrophomonas* isolates recovered from effluent samples and veterinary hospitalization units. The species identified included *S. maltophilia*, *S. hibiscicola*, *S. muris*, and *S. forensis*, with 13 distinct sequence types (STs) detected, including ST1402, which was shared between both environments. Antimicrobial susceptibility testing revealed a high resistance profile to ciprofloxacin, whereas resistance to tetracyclines, chloramphenicol, levofloxacin, and trimethoprim/sulfamethoxazole was less frequent. In accordance with the reported intrinsic resistance of *Stenotrophomonas* to a range of antibiotic classes, the strains were resistant to meropenem, ampicillin, cefoxitin, and amoxicillin/clavulanate. Whole-genome sequencing confirmed the universal presence of intrinsic β-lactamase genes *blaL1* and *blaL2*, indicating the genetic potential for β-lactamases production, as well as the widespread distribution of RND-type efflux pumps, including components of the SmeABC and SmeDEF systems. In addition, all isolates were strong biofilm formers. Together, these findings indicate that environmental *Stenotrophomonas* spp. strains from veterinary settings harbor multidrug resistance determinants, efflux-mediated mechanisms, and biofilm-forming capacity, highlighting their potential role in the maintenance and dissemination of clinically relevant antimicrobial resistance.

**Supplementary Information:**

The online version contains supplementary material available at https://doi.org/10.1007/s11259-026-11539-z.

## Introduction

The genus *Stenotrophomonas* has gained prominence due to its clinical relevance and impact on public health. Among its species, *Stenotrophomonas maltophilia* stands out as an emerging opportunistic pathogen, frequently associated with infections in immunocompromised patients (Brooke 2012). In addition to its clinical role, this species exhibits a broad environmental distribution, being isolated from soil, water, plants, and from hospital surfaces (An and Berg 2018).

The resistance of *S. maltophilia* to multiple classes of antimicrobials results from a combination of intrinsic mechanisms, such as low outer membrane permeability and efflux pump systems (Gil-Gil et al. 2020), with acquired resistance determinants frequently associated with mobile genetic elements, such as integrons, plasmids, and transposons (Furlan et al. 2019). This diversity of resistance strategies includes, for example, the inactivation of β-lactams (Mojica et al. 2022), which directly limits therapeutic options. Intrinsic resistance in this species notably affects several β-lactams, particularly carbapenems, as well as aminoglycosides (Mojica et al. 2022).

In the context of human infections, the treatment of *S. maltophilia* is particularly challenging. Trimethoprim–sulfamethoxazole (TMP-SMX) remains the first-line therapy, although cases of resistance have been widely described (Kim et al. 2024). Therapeutic alternatives, such as fluoroquinolones and tetracyclines, show variable efficacy, which is often associated with resistance profiles specific to clinical or environmental niches (Brooke 2012; Mojica et al. 2022).

Beyond human infections, *S. maltophilia* has also been isolated from veterinary contexts, including domestic animals (Shimizu et al. 2021) and livestock (Ohnishi et al. 2012), where it has been associated with opportunistic infections similar to those described in humans. These strains frequently exhibit resistance profiles comparable to those observed in the human clinical context, suggesting the possible existence of animal reservoirs and reinforcing the connection between human, veterinary, and environmental health.

In addition, environmentally distributed *Stenotrophomonas* spp. also have the potential to develop or acquire antimicrobial resistance (Ortiz Álvarez et al. 2025), increasing the challenges for infection control. In this context, *Stenotrophomonas muris*, recently described and already associated with opportunistic infections in humans (Liu et al. 2025), stands out, as well as *Stenotrophomonas hibiscicola*, isolated from plants (Nguyen et al. 2024), and *Stenotrophomonas forensis*, initially described as a contaminant in viral transport media (Nguyen et al. 2024), reinforcing the importance of bacterial surveillance in different contexts.

Considering this scenario, this study aimed to characterize the genetic diversity of *Stenotrophomonas* and evaluate their potential for the spread of antimicrobial resistance and virulence-associated traits veterinary hospital environments.

## Materials and methods

### Sampling and bacterial strains isolation

Bacterial strains were obtained from environmental surface samples collected at a Veterinary Teaching Hospital over a six-month period (July 2023 to March 2024), including dog hospitalization units (DHU), cat hospitalization units (CHU), and hospital wastewater (HW).

Samples were collected weekly from the hospitalization units (DHU and CHU) using sterile swabs, and further incubated in brain heart infusion (BHI) broth supplemented with meropenem (1 µg/mL) at 37 °C for 24 h, followed by plating onto MacConkey agar supplemented with meropenem (MCM) (1 µg/mL; Sigma-Aldrich, USA). After incubation at 37 °C for 24 h, lactose-non-fermenting colonies were selected and subcultured on Luria-Bertani agar (LB; Sigma-Aldrich, USA) and incubated at 37 °C for 24 h (Tartor et al. 2024). A total of 280 samples were collected from the hospitalization units, including 174 from the DHU and 106 from CHU.

Additionally, HW samples were collected biweekly from the sewage system of the hospital. A volume of 50 mL from each sample was vacuum-filtered through sterile 0.22-µm membrane filters. The membranes were transferred to tubes containing 10 mL of 0.85% saline solution, corresponding to the initial dilution. Serial dilutions (10^−^¹ to10^−^³) were prepared and plated onto MCM and incubated at 37 °C for 24 h. Lactose-non-fermenting colonies were then selected and subcultured on LB agar and incubated at 37 °C for 24 h. A total of 28 HW samples were collected during the study period.

All isolates were identified by matrix-assisted laser desorption/ionization time-of-flight mass spectrometry (MALDI-TOF MS; Bruker). Only isolates identified as *Stenotrophomonas* spp. were included in the following analyses. A total of 17 *Stenotrophomonas* isolates were recovered and further characterized.

### Genomic DNA extraction of *Stenotrophomonas* spp.

Genomic DNA was extracted from all *Stenotrophomonas* spp. strains isolated in this study. The isolates were subcultured on tryptic soy agar (TSA; Merck, Darmstadt, Germany) and subsequently inoculated into tryptic soy broth (TSB; Merck, Darmstadt, Germany), followed by incubation at 37 °C overnight. A 3 mL aliquot of each bacterial culture was centrifuged at 18,407 × g for 5 min to obtain a cell pellet. The supernatant was discarded, and genomic DNA was extracted from the pellet using the QIAamp^®^ DNA Mini Kit (Qiagen, Hilden, Germany), according to the manufacturer’s instructions. DNA quality and quantity were determined using a NanoDrop One^C^ (Thermo Fisher Scientific, USA) and the Qubit dsDNA BR assay (Thermo Fisher Scientific, USA), respectively.

### Whole-genome sequencing and genomic analyses

DNA libraries were prepared and sequenced using the Illumina NovaSeq 6000 platform with 2 × 150 bp paired-end, according to the manufacturer’s instructions (Illumina, San Diego, CA, USA). The raw reads quality was assessed using FastQC 0.11.9 (Andrews 2010), then adapter, short-length (> 150 nt) and low-quality (Phred ≥ 33) sequences were trimmed using Trimmomatic 0.39 (Bolger et al. 2014). Genome assembly was performed using SPAdes (Prjibelski et al. 2020). The identification and segregation of plasmid sequences were carried out using PlasmidFinder 2.1 (Carattoli et al. 2014). Afterward, genome annotation was performed using Prokka 1.14.6. (Seemann 2014). Antimicrobial resistance genes were identified by ResFinder 4.7.2 (Florensa et al. 2022) and the Comprehensive Antibiotic Resistance Database (Alcock et al. 2020). Virulence genes were identified using the VirulenceFinder 2.0 (Kleinheinz et al. 2014).

Sequence types (STs) of the *Stenotrophomonas* spp. isolates were determined using the multilocus sequence typing (MLST) scheme available in the PubMLST database (accessed on January 20, 2026).

### Phenotypic characterization of *Stenotrophomonas* spp.

#### In vitro biofilm formation evaluation

In vitro biofilm formation was evaluated with minor modifications, as previously described (Gallo et al. 2017).The isolates were subcultured on TSA and incubated at 37 °C for 24 h. For biofilm formation, TSB was used, and the absorbance was measured at 550 nm (OD_550_) in a Multiskan FC microplate photometer (Thermo Fischer Scientific, Massassuchets, USA).

Based on the absorbance, the strains were classified into the following categories: non-biofilm producer (OD ≤ ODc-), weak biofilm producer (ODc- < OD ≤ 2 x ODc-), moderate biofilm producer (2 x ODc- < OD ≤ 4 x ODc-) and strong biofilm producer (4 x ODc- < OD), as proposed by (STEPANOVIĆ et al., 2007). Three biological replicates were performed for each strain, with four technical replicates. *Acinetobacter baumannii* ATCC 17,978 was used as a positive control, while wells containing only TBS broth were used as negative controls.

#### Curli and cellulose production evaluation

Curli and cellulose potential production were evaluated according to the methods previously described, with some modifications (Solomon et al. 2005). Because these phenotypic assays have been primarily validated in Enterobacterales, the results were interpreted as extracellular matrix-associated phenotypes consistent with, but not conclusive evidence of, curli- and cellulose-like structures. Morphotype classifications were performed in biological and technical duplicates.

To determine curli expression, the strains were spot-inoculated on no-salt LB agar supplemented with Congo Red (100 mg/L) and Brilliant Blue (20 mg/L) (Sigma-Aldrich, Saint Louis, MO, USA), referred to as LB-CRBB, and incubated at 37 °C for 48 h and at 28 °C for 48 h. Morphotypes were classified as *rdar* (violet, dry, and rough), indicating production of both curli and cellulose; *bdar* (brown, dry, and rough), indicating production of curli and absence of cellulose; and *saw* (smooth and white), indicating absence of both components (Bokranz et al. 2005). In addition to the classical morphotypes, less pronounced forms corresponding to intermediate expression of curli and/or cellulose were also considered, described as *ras* (violet and smooth), *bas* (brown and smooth), and *pas* (pink and smooth) (Bokranz et al. 2005).

Cellulose production was evaluated by spotting on no-salt LB agar containing calcofluor (200 mg/L; Fluorescent Brightener 28, Sigma-Aldrich, Saint Louis, MO, USA), with incubation at both 37 °C for 48 h and 28 °C for 48 h, followed by exposure to UV light (366 nm) (Solomon et al. 2005).

#### Determination of minimum inhibitory concentration for meropenem

Minimum inhibitory concentrations (MICs) of meropenem against the *Stenotrophomonas* spp. strains were determined using the broth microdilution method, according to Clinical and Laboratory Standards Institute guidelines (CLSI M07 2024) and as previously described by (da Silva et al. 2025), using meropenem concentrations ranging from 0.5 to 128 µg/mL. *Pseudomonas aeruginosa* ATCC 27,853 was used as a reference strain. MIC results were defined as the lowest concentration of meropenem that inhibited bacterial growth. All tests were performed in technical triplicates. Interpretation of the results was performed using the CLSI VET01S clinical breakpoints established for *P. aeruginosa* (CLSI 2024), in which isolates with MIC values ≥ 8 µg/mL were considered resistant.

#### Disk diffusion antimicrobial susceptibility testing

Antimicrobial susceptibility of the *Stenotrophomonas* spp. strains was also assessed to a set of 13 antimicrobial agents, representing eight antibiotic classes: aminoglycosides (amikacin [AMK] 30 µg, gentamicin [GEN] 10 µg, tobramycin [TOB] 10 µg), amphenicols (chloramphenicol [C] 30 µg), cephalosporins (ceftazidime [CAZ] 30 µg, cefoxitin [CFO] 30 µg), fluoroquinolones (levofloxacin [LVX] 5 µg, ciprofloxacin [CIP] 5 µg), folate pathway inhibitors (trimethoprim/sulfamethoxazole [TMP-SMX] 23.75 µg), tetracyclines (doxycycline [DOX] 30 µg, tetracycline [TET] 30 µg), penicillin (ampicillin [AMP] 10 µg), and beta-lactamase inhibitor penicillin (amoxicillin/clavulanate [AMC] 30 µg). This analysis was performed by agar disk diffusion, following CLSI M02 guidelines (CLSI 2015). *Escherichia coli* ATCC 25,922 was used as the quality control strain. Susceptibility results were interpreted according to the CLSI M100 (CLSI 2025) and VET01S (CLSI 2024) (Table S1). When *Stenotrophomonas*-specific CLSI breakpoints were unavailable, interpretative criteria from other bacterial groups were applied, being the corresponding reference organisms indicated in Table S1.

### Phylogenetic analysis

Two phylogenetic approaches were employed to investigate the genetic relationships among the isolates from this study, based on the 16 S rRNA gene and housekeeping genes (*atpD*,* gapA*,* guaA*,* mutM*,* nuoD*,* ppsA*, and *recA*). The 16 S rRNA gene sequence was aligned in MEGA v.12.0.11 (Kumar et al. 2024) using the ClustalW algorithm. For the multilocus analysis, the sequences of the seven housekeeping genes were individually aligned with the MUSCLE algorithm in the same software, and poorly aligned regions were removed using Gblocks v.0.91.1 (Talavera and Castresana 2007). The filtered alignments were concatenated in MEGA and used for phylogenetic reconstruction using the neighbor-joining method with 10,000 bootstrap replicates, applying the Tamura 3-parameter model for the 16 S rRNA gene and the Tamura–Nei model for the concatenated housekeeping genes. The reference genomes *Pseudomonas putida* NBRC 14,164 (access NC_021505.1), *P. putida* IAM 1236 (access NR_043424.1), *S. muris* DSM 28,631 (access GCA_024621935.1), and *S. maltophilia* NCTC10257 (access LT906480.1) was used in the phylogenetic reconstruction.

## Results

### Bacterial identification, whole-genome sequencing and assembly metrics

A total of 17 *Stenotrophomonas* spp. environmental strains were identified in the veterinary hospital facilities over a six-month period (July 2023 to March 2024), being recovered from 280 sampled hospitalization units (174 DHU and 106 CHU) and 28 samples from HW. In DHU and CHU, *S. maltophilia* (*n* = 7), *S. muris* (*n* = 2), and *S. hibiscicola* (*n* = 1) were identified; whereas *S. maltophilia* (*n* = 4), *S. forensis* (*n* = 2), and *S. hibiscicola* (*n* = 1) were identified in HW (Table 1).

**Table 1:** General information on the bacterial strains in the present study

Strain ID	Isolation source	Bacterial species	ST	Collection date
D1	DHU	*Stenotrophomonas maltophilia*	1403	07/31/2023
D2	DHU	*Stenotrophomonas muris*	930	07/31/2023
D3	DHU	*Stenotrophomonas muris*	300	09/11/2023
D4	DHU	*Stenotrophomonas maltophilia*	950	09/18/2023
D5	DHU	*Stenotrophomonas maltophilia*	1407	11/20/2023
D6	DHU	*Stenotrophomonas maltophilia*	1407	11/20/2023
D7	DHU	*Stenotrophomonas hibiscicola*	1402	12/18/2023
D8	DHU	*Stenotrophomonas maltophilia*	1399	01/22/2024
D9	DHU	*Stenotrophomonas maltophilia*	1399	01/29/2024
C1	CHU	*Stenotrophomonas maltophilia*	1405	03/27/2024
W1	HW	*Stenotrophomonas maltophilia*	212	08/21/2023
W2	HW	*Stenotrophomonas maltophilia*	212	08/21/2023
W3	HW	*Stenotrophomonas forensis*	1404	08/21/2023
W4	HW	*Stenotrophomonas forensis*	1404	09/10/2023
W5	HW	*Stenotrophomonas maltophilia*	770	09/10/2023
W6	HW	*Stenotrophomonas maltophilia*	1400	01/29/2024
W7	HW	*Stenotrophomonas hibiscicola*	1402	02/05/2024

DHU = dog hospitalization unit; CHU = cat hospitalization unit; HW = hospital wastewater

The estimated sequencing depth of the genomes ranged from 298 × to 438 ×, ensuring sufficient coverage for high-quality downstream analyses. *De novo* assemblies resulted in 45–89 contigs per strain, and N50 values ranging from 100,039 to 256,315 bp. The assembled genome sizes varied between 4,314,025 and 4,871,521 pb. General information regarding genome assembly was described in Table S2, including the access number to the genomes on NCBI database.

Regarding STs, 13 distinct STs were identified among the strains, of which eight were recovered from hospitalization units (ST300, ST930, ST950, ST1399, ST1402, ST1403, ST1405, and ST1407) and five from effluent samples (ST212, ST770, ST1400, ST1402, and ST1404). ST1402 was the only ST shared between both environments (D7 and W7 *S. hibiscicola*) (Table S2).

The genomic antimicrobial resistance profiles revealed a diverse set of genes associated with antibiotic inactivation, efflux-mediated resistance, and target replacement across the analyzed *Stenotrophomonas* strains (Table 2). The *adeF* gene, encoding a component of a resistance-nodulation-division (RND)-type efflux pump, was detected in all strains. Efflux-related genes of the Sme system were widely distributed among the strains, including components of the SmeABC (*smeA*,* smeB*,* smeC*) and SmeDEF (*smeD*,* smeE*,* smeF*) efflux pump systems. The regulatory genes *smeR* and *smeS* were also identified in most strains.

**Table 2:** Antimicrobial resistance genes and genes associated with adhesion and environmental persistence predicted by whole-genome sequencing analyses

Strain ID	Isolation source	Bacterial species	ST	Resistance Gene Profiles							Adhesion and environmental persistence-associated genes	
				Aminoglycosides	β-lactams	Multidrug efflux	Sulfonamides	Tetracyclines	Glycopeptides	Disinfectants/ antiseptics	Adhesion	Regulation
D1	DHU	*S. maltophilia*	1403	*aac(6’)-Iz; aph(3’)-IIc; aph(9)-Ic*	*blaL1; blaL2*	*adeF; smeA; smeB; smeC; smeD; smeE; smeF; smeS*,* smeR*	-	-	-	*qacJ*	*tufA*; *smf*-1; *spgM*; *rmlA*; *rmlC*; *xanB*	*csrA*
D2	DHU	*S. muris*	930	*aph(9)-Ic*	*blaL1; blaL2*	*adeF; smeB; smeD; smeE; smeF; smeR*	-	-	-	*qacJ*	*tufA*; *smf*-1; *spgM*; *rmlA*; *rmlC*	*csrA*
D3	DHU	*S. muris*	300	*aph(9)-Ic*	*blaL1; blaL2*	*adeF; smeB; smeD; smeE; smeF; smeR*	-	-	-	*qacJ*	*tufA*; *smf*-1; *spgM*; *rmlA*; *rmlC*	*csrA*
D4	DHU	*S. maltophilia*	950	*aph(9)-Ic*	*blaL1; blaL2*	*adeF; smeB; smeC; smeD; smeE; smeF; smeR; smeS*	-	-	-	*qacJ*	*tufA*; *smf*-1; *spgM*; *rmlA*; *rmlC*	*csrA*
D5	DHU	*S. maltophilia*	1407	*aac(6’)-Iz; aph(3’)-IIc; aph(9)-Ic*	*blaL1; blaL2*	*adeF; smeA; smeB; smeC; smeD; smeE; smeF; smeR; smeS*	-	-	-	*qacG*	*tufA*; *smf*-1; *spgM*; *rmlA*; *rmlC*; *xanB*	*csrA*
D6	DHU	*S. maltophilia*	1407	*aac(6’)-Iz; aph(3’)-IIc; aph(9)-Ic*	*blaL1; blaL2*	*adeF; smeA; smeB; smeC; smeD; smeE; smeF; smeR; smeS*	-	-	-	*qacG*	*tufA*; *smf*-1; *spgM*; *rmlA*; *rmlC*; *xanB*	*csrA*
D7	DHU	*S. hibiscicola*	1402	*aph(9)-Ic*	*blaL1; blaL2*	*adeF; smeD; smeE; smeF;*	-	-	-	*qacG*	*tufA*; *smf*-1; *spgM*; *rmlA*; *rmlC*	*csrA*
D8	DHU	*S. maltophilia*	1399	*aac(6’)-Iz; aph(3’)-IIc*	*blaL1; blaL2*	*adeF; smeA; smeB; smeC; smeD; smeE; smeF; smeR; smeS*	-	-	-	*qacJ*	*tufA*; *smf*-1; *spgM*; *rmlA*; *rmlC*; *xanB*	*csrA*
D9	DHU	*S. maltophilia*	1399	*aac(6’)-Iz; aph(3’)-IIc; aph(3’)-IIc; aph(9)-Ic*	*blaL1; blaL2*	*adeF; smeA; smeB; smeC; smeD; smeE; smeF; smeR; smeS*	-	-	-	*qacJ*	*tufA*; *smf*-1; *spgM*; *rmlA*; *rmlC*; *xanB*	*csrA*
C1	CHU	*S. maltophilia*	1405	*aph(9)-Ic*	*blaL1; blaL2*	*adeF; smeB; smeC; smeD; smeE; smeF; smeR; smeS*	-	-	-	*qacJ*	*tufA*; *smf*-1; *spgM*; *rmlA*; *rmlC*	*csrA*
W1	HW	*S. maltophilia*	212	*aac(6’)-Iib; aph(3’)-IIc; aph(9)-Ic; aadA7*	*blaL1; blaL2*	*adeF; smeA; smeB; smeC; smeD; smeE; smeF; smeR; smeS*	*sul1*	-	-	*qacG*	*tufA*; *smf*-1; *spgM*; *rmlA*; *rmlC*	*csrA*
W2	HW	*S. maltophilia*	212	*aac(6’)-Iib; aph(3’)-IIc; aph(9)-Ic; aadA7*	*blaL1; blaL2*	*adeF; smeA; smeB; smeC; smeD; smeE; smeF; smeR; smeS*	*sul1*	-	-	*qacG*	*tufA*; *smf*-1; *spgM*; *rmlA*; *rmlC*	*csrA*
W3	HW	*S. forensis*	1404	*aac(6’)-Ib3; aac(6’)-Ib9; aph(9)-Ic; aadA5*	*blaL1; blaL2*	*adeF; smeB; smeD; smeE; smeF; smeR*	*sul1*	-	-	*qacJ*	*tufA*; *smf*-1; *spgM*; *rmlA*; *rmlC*	*csrA*
W4	HW	*S. forensis*	1404	*aac(6’)-Ib’; aac(6’)-Ib3; aph(9)-Ic; aadA4*	*blaL1; blaL2*	*adeF; smeB; smeD; smeE; smeF; smeR*	*sul1*	-	-	*qacJ*	*tufA*; *smf*-1; *spgM*; *rmlA*; *rmlC*	*csrA*
W5	HW	*S. maltophilia*	770	*aac(6’)-Iz; aph(3’)-IIc; aph(3’)-IIa; aph(3’’)-Ib; aph(6)-Id; aph(6)-Ic; aph(9)-Ic*	*blaL1; blaL2*	*adeF; smeA; smeB; smeC; smeD; smeE; smeF; smeR; smeS*	*sul2*	*tet(G)*	*BLMT*	*qacJ*	*tufA*; *smf*-1; *spgM*; *rmlA*; *rmlC*; *xanB*	*csrA*
W6	HW	*S. maltophilia*	1400	*aac(6’)-Iz; aph(9)-Ic; aph(3’)-IIc*	*blaL1; blaL2*	*adeF; smeA; smeB; smeC; smeD; smeE; smeF; smeR; smeS*	*-*	-	-	*qacG*	*tufA*; *smf*-1; *spgM*; *rmlA*; *rmlC*; *xanB*	*csrA*
W7	HW	*S. hibiscicola*	1402	*aph(3’)-IIc; aph(9)-Ic*	*blaL1; blaL2*	*adeF; smeD; smeE; smeF*	*-*	-	-	*qacG*	*tufA*; *smf*-1; *spgM*; *rmlA*; *rmlC*	*csrA*

- = gene absent; DHU = dog hospitalization unit; CHU = cat hospitalization unit; HW = hospital wastewater

Aminoglycoside resistance genes were widely detected, comprising multiple variants of aminoglycoside-modifying enzymes, including members of the AAC (aminoglycoside acetyltransferases), APH (aminoglycoside phosphotransferases), and AAD (aminoglycoside nucleotidyltransferases) families, with frequent co-occurrence within the same strains. Also, the intrinsic β-lactamase genes *blaL1* and *blaL2* were identified in all strains, encoding class B and class A β-lactamases, respectively (Table 2).

Genes associated with disinfectant resistance (*qacJ* or *qacG*) were detected in all strains (Table 2). Additionally, target replacement genes related to sulfonamide resistance (*sul1* and *sul2*) were found in five effluent-derived strains (W1–W5). The tetracycline resistance gene *tet(G)* and the bleomycin resistance gene *BLMT* were identified exclusively in strain W5.

In addition to resistance determinants, all strains harbored genes associated with adhesion and environmental persistence (Table 2). The detected genetic repertoire was predominantly related to environmental maintenance and colonization traits. In summary, we detected adhesion-related genes, including *tufA*, *smf-1*, *spgM*, *rmlA*, *rmlC*, and *xanB*, as well as the global regulatory gene *csrA*, which was present in all strains.

### Assessment of the phenotypic profile of the *Stenotrophomonas* strains

#### Classification of strains according to multidrug resistance criteria

The in vitro analysis of antimicrobial susceptibility revealed resistance to multiple antimicrobial classes among the *Stenotrophomonas* spp. strains. Based on the criteria proposed by Magiorakos et al. (2012), which define multidrug resistance as non-susceptibility to at least one agent in three or more antimicrobial categories, all isolates fulfilled the operational phenotypic MDR criterion under the interpretive framework applied in this study. However, this classification should be interpreted with caution, as part of the observed resistance profile, particularly to β-lactam agents and aminoglycosides, is attributable to intrinsic resistance in *Stenotrophomonas maltophilia*. In addition, because *Stenotrophomonas*-specific breakpoints for in vitro antimicrobial susceptibility testing were unavailable for most of the antimicrobial agents (Table S1), the corresponding interpretations do not necessarily represent clinically validated resistance categories for this genus.

Despite the prudence in the phenotypic antimicrobial susceptibility interpretation, as little is known about the *Stenotrophomonas* profile, especially non-maltophilia strains, it is noteworthy the observation of all strains (17/17) were resistant to both ampicillin and cefoxitin, while resistance to amoxicillin/clavulanate and ciprofloxacin was detected in the majority of strains (Table 3). Resistance to aminoglycosides and cephalosporins was observed in a high proportion of strains, whereas resistance to tetracyclines, amphenicols, fluoroquinolones (except ciprofloxacin), and sulfonamides was detected in only a few strains (Table 3; Table S3).

**Table 3:** In vitro antimicrobial resistance profile of Stenotrophomonas spp

Antimicrobial classes	Antimicrobial agent	Tested Method	Resistant strains
Aminoglycosides*	Amikacin	Disk diffusion	47%; 8/17
	Gentamicin	Disk diffusion	53%; 9/17
	Tobramycin	Disk diffusion	59%; 10/17
Cephalosporins	Ceftazidime	Disk diffusion	41%; 7/17
	Cefoxitin	Disk diffusion	100%; 17/17
Amphenicols	Chloramphenicol	Disk diffusion	6%; 1/17
Fluoroquinolones	Levofloxacin	Disk diffusion	6%; 1/17
	Ciprofloxacin	Disk diffusion	76%; 13/17
Folate pathway inhibitors	Trimethoprim/sulfamethoxazole	Disk diffusion	23%; 4/17
Tetracyclines	Doxycycline	Disk diffusion	6%; 1/17
	Tetracycline	Disk diffusion	12%; 2/17
Penicillins*	Ampicillin	Disk diffusion	100%; 17/17
β-lactamase inhibitor combinations	Amoxicillin/clavulanate*	Disk diffusion	94%; 16/17
Carbapenems*	Meropenem	MIC	100%; 17/17

*Resistance to these antimicrobial classes is considered intrinsic in *Stenotrophomonas maltophilia* according to CLSI M100, 36th Edition (2026)

Notably, all *Stenotrophomonas* strains showed meropenem MIC values above the interpretive criteria adopted in this study (Table 3; Table S3).

Remarkable, *S. maltophilia* W5 was the only strain resistant to doxycycline, levofloxacin, and chloramphenicol, whereas all remaining strains were susceptible to these antimicrobials (Table S3). Resistance to trimethoprim/sulfamethoxazole was observed exclusively among effluent strains, including *S. maltophilia* W2 and W5, and *S. forensis* W3 and W4.

#### Adhesion-associated phenotypic traits in *Stenotrophomonas* spp. strains

Biofilm formation and adherence-related traits were in vitro evaluated among the investigated strains (Table 4). Importantly, all investigated strains were classified as strong biofilm producers. All strains exhibited a bas/bas morphotype at both 37 °C and 28 °C, which is commonly associated with curli expression, while calcofluor binding was detected under all incubation conditions, suggesting the presence of cellulose-like extracellular matrix components (Table 4; Figure S1). However, these phenotypes are considered indicative of curli- and cellulose-like traits rather than direct evidence of their production in *Stenotrophomonas*, as the used assays were primarily developed for Enterobacterales.

**Table 4:** Adhesion-associated phenotypic traits of the Stenotrophomonas spp. strains

Strain ID	Bacterial species	Morphotype on LB-CRBB plates (at 28 °C/37 °C) – curli-associated phenotype			Calcofluor binding (at 28 °C/37 °C) – cellulose-like phenotype	Biofilm Formation		
D1	*S. maltophilia*	bas/bas			+/+	Strong		
D2	*S. muris*	bas/bas			+/+	Strong		
D3	*S. muris*	bas/bas			+/+	Strong		
D4	*S. maltophilia*	bas/bas			+/+	Strong		
D5	*S. maltophilia*	bas/bas			+/+	Strong		
D6	*S. maltophilia*	bas/bas			+/+	Strong		
D7	*S. hibiscicola*	bas/bas			+/+	Strong		
D8	*S. maltophilia*	bas/bas			+/+	Strong		
D9	*S. maltophilia*	bas/bas			+/+	Strong		
C1	*S. maltophilia*	bas/bas			+/+	Strong		
W1	*S. maltophilia*	bas/bas			+/+	Strong		
W2	*S. maltophilia*	bas/bas			+/+	Strong		
W3	*S. forensis*	bas/bas			+/+	Strong		
W4	*S. forensis*	bas/bas			+/+	Strong		
W5	*S. maltophilia*	bas/bas			+/+	Strong		
W6	*S. maltophilia*	bas/bas			+/+	Strong		
W7	*S. hibiscicola*	bas/bas			+/+	Strong		

bas = brown and smooth colony morphology; += positive calcofluor-binding phenotype

### Multilocus phylogeny reveals clear species-level structure in *Stenotrophomonas* spp. strains

**Fig. 1:**
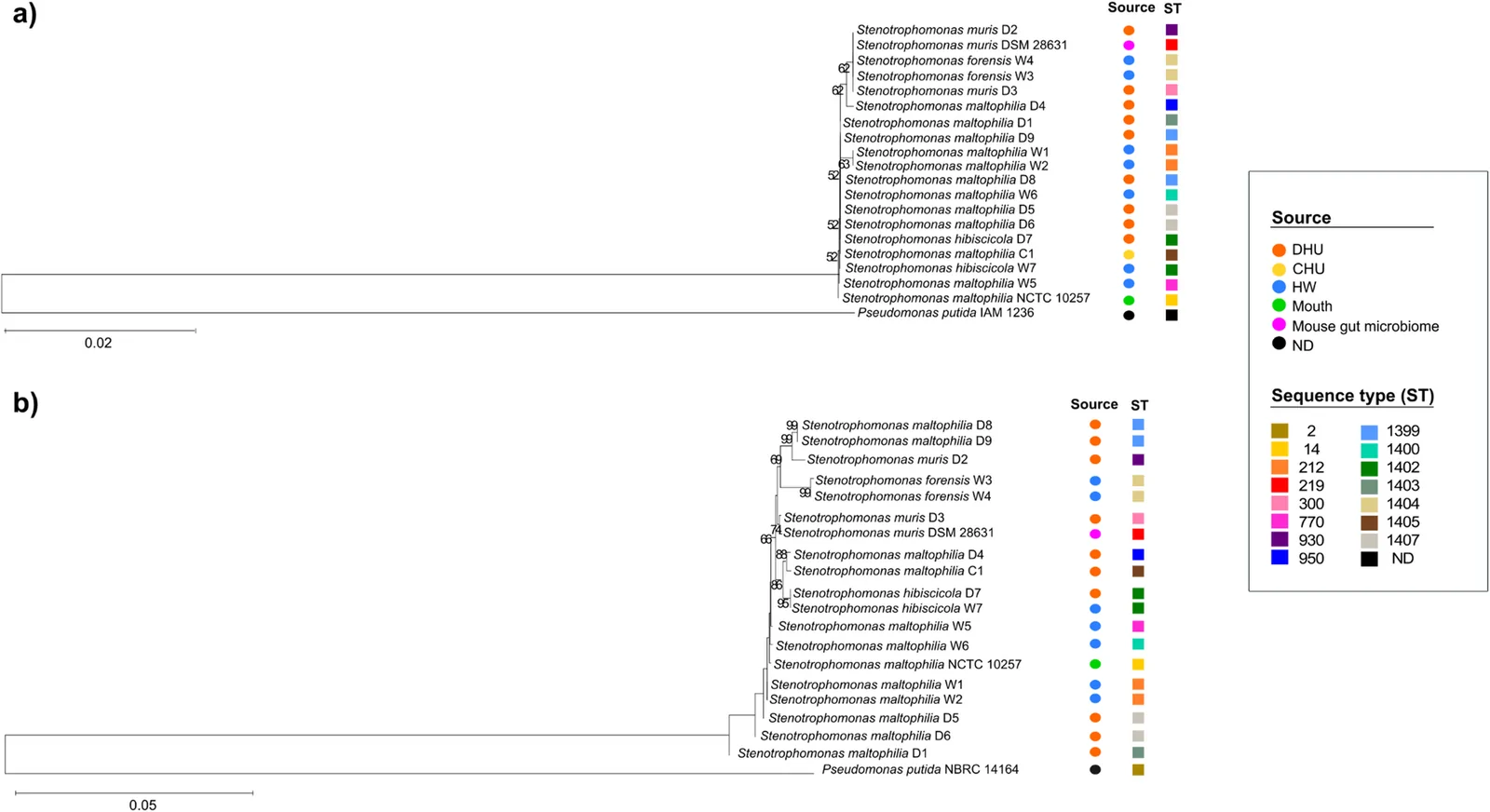
Phylogenetic relationships among *Stenotrophomonas* spp. strains, including *S. maltophilia*, *S. muris*, *S. hibiscicola* and *S. forensis*. (**a**) 16 S rRNA neighbor-joining tree (bootstrap = 10,000) constructed using the Tamura 3-parameter model (**b**) Neighbor-joining tree based on concatenated housekeeping genes (*atpD*, *gapA*, *guaA*, *mutM*, *nuoD*, *ppsA*, and *recA*), constructed using the Tamura–Nei model (Gamma = 0.16) with 10,000 bootstrap replicates. *P. putida* was used as the outgroup in both analyses. DHU = dog hospitalization unit; CHU = cat hospitalization unit; HW = hospital wastewater; ND = not determined; ST = sequence typing

Phylogenetic relationships among the strains were inferred using both the 16 S rDNA gene and a concatenated set of housekeeping genes. The phylogenetic tree based on the 16 S rDNA gene (Fig. 1A) showed limited discriminatory power among the analyzed strains. Strains assigned to different *Stenotrophomonas* species frequently clustered together, resulting in broad clades with poor species-level resolution. *S. muris*, *S. forensis*, and *S. hibiscicola* were interspersed within the same clades.

In contrast, the phylogenetic analysis based on concatenated housekeeping genes (Fig. 1B) showed clearer species-level clustering among the analyzed strains. Strains grouped according to their respective species, with *S. maltophilia*, *S. muris*, *S. forensis*, and *S. hibiscicola* forming well-defined clades supported by higher bootstrap values. These results indicate that, in the present dataset, concatenated housekeeping genes provided higher species-level resolution than 16 S rDNA gene analysis.

As shown in Fig. 1B, phylogenetic clustering reflected both the source of isolation and the assigned STs. As the strains were from the same hospital, during a longitudinal collection period, the phylogenetic profile indicates the presence of multiple genetically distinct lineages within the analyzed environment.

## Discussion

This study demonstrates that environmental *Stenotrophomonas* spp. strains originating from a veterinary hospital exhibit remarkable genetic diversity and resistance to clinically important antimicrobials. Although part of this resistance is intrinsic to the genus, these inherent mechanisms may contribute to the persistence of antimicrobial resistance traits in bacterial populations, highlighting the potential role of environmental *Stenotrophomonas* strains as reservoirs of resistance to clinically important antimicrobials.

Different *Stenotrophomonas* species were recovered from hospitalization units and hospital wastewater. *S. muris* was exclusively detected in hospitalization units, whereas *S. forensis* was restricted to hospital wastewater. In contrast, *S. maltophilia* predominated across multiple environments. *S. hibiscicola* was also identified in both settings, with one strain recovered from each. Phylogenetic analyses based on housekeeping genes provided greater species-level resolution than 16 S rRNA gene analysis, allowing clear discrimination among the investigated *Stenotrophomonas* species. Notably, the presence of *S. hibiscicola*, a species originally associated with plants (Nguyen et al. 2024), in both hospitalization units and effluents suggests ecological flexibility and adaptive potential.

High ST diversity was observed among the environmental *Stenotrophomonas* strains, in agreement with previous MLST-based studies reporting extensive genetic heterogeneity within this genus, including analyses based on clinical strains (Nicolas-sayago et al. 2025). The identification of ST1402, corresponding to *S. hibiscicola*, in both hospitalization units and effluent samples suggests that this sequence type may occur across different hospital-associated environments. This finding is consistent with reports describing identical STs in both clinical and environmental settings within human hospital contexts (Emami et al. 2022).

In this context, in vitro resistance to trimethoprim/sulfamethoxazole, observed in 23% of the environmental strains, deserves attention, as this antimicrobial remains the primary therapeutic option recommended for human infections caused by *Stenotrophomonas* spp. Notably, the resistant strains – *S. maltophilia* W2, *S. maltophilia* W5, *S. forensis* W3, and *S. forensis* W4 were recovered from effluent samples, highlighting their potential role as reservoirs of resistant bacteria. From a genotypic perspective, these strains harbored both *sul1* and/or *sul2* genes, which are associated with sulfonamide resistance, aligning with the observed phenotypic resistance profile. Moreover, the trimethoprim/sulfamethoxazole resistance rate observed in this study is consistent with those reported for human clinical *S. maltophilia* strains, with rates of up to 28% reported in a previous analyses of strains collected between 2005 and 2022 (Kim et al. 2024). In contrast, studies based on veterinary clinical samples have reported significantly higher proportions of trimethoprim/sulfamethoxazole resistance, reaching up to 66% in clinical strains from dogs and cats (Shimizu et al. 2021).

Notably, wastewater-derived *Stenotrophomonas* spp. strains encode resistance markers to a broad spectrum of antimicrobial compounds, including disinfectants and antiseptics. These findings underscore the potential role of aquatic systems as reservoirs and possible dissemination routes for antimicrobial and antiseptic resistance determinants.

Genes encoding RND-type efflux pumps were detected in all strains analyzed in the present study. Components of the SmeDEF system (*smeD*, *smeE*, *smeF*) were universally present, whereas elements of the SmeABC system (*smeA*, *smeB*, *smeC*) showed variable distribution. Overexpression of SmeDEF has been associated with reduced susceptibility to fluoroquinolones (Zhang et al. 2001), while overexpression of SmeABC contributes to decreased susceptibility to fluoroquinolones, β-lactams, and aminoglycosides (Cho et al. 2012). The widespread presence of these intrinsic efflux determinants in strains from effluents and veterinary hospitalization units may contribute to antimicrobial tolerance in these environments.

Among the evaluated strains, in vitro resistance to chloramphenicol, levofloxacin, and doxycycline was observed at a low frequency (6%, 1/17 for each drug), restricted to strain *S. maltophilia* W5. Phenotypic resistance to tetracycline was detected in two strains (*S. maltophilia* W5 and *S. hibiscicola* W7). Genomic analysis revealed the presence of the tetracycline resistance gene *tet(G)*, a determinant commonly associated with acquired antimicrobial resistance, exclusively in *S. maltophilia* W5, whereas no *tet* genes were identified in *S. hibiscicola* W7. The detection of tetracycline resistance in *S. hibiscicola* W7 despite the absence of identifiable *tet* determinants highlights the complexity of antimicrobial resistance in this genus. Given the known limitations in susceptibility testing standardization for *Stenotrophomonas* spp., these findings should be interpreted with caution. Overall, resistance to these agents was observed at low frequency among the evaluated environmental strains, suggesting that resistance to these antimicrobials remains limited in the analyzed population.

The high meropenem MIC values observed in all strains are consistent with the well-characterized intrinsic β-lactam resistance of *S. maltophilia* (Yang et al. 2014), which is mainly mediated by the *blaL1* gene – a class B metallo-β-lactamase with a broad substrate spectrum including carbapenems –, and the *blaL2* gene – a class A serine β-lactamase contributing to resistance against other β-lactams (Yang et al. 2014). According to our genomic analyses, both genes were detected in all studied strains, indicating their genetic potential to produce these β-lactamases.

All *Stenotrophomonas* spp. strains in this study were classified as strong biofilm producers and exhibited phenotypes potentially associated with curli- and cellulose- like extracellular matrix components, highlighting the phenotypic plasticity of this genus and its potential for environmental persistence. Accordingly, the ability to produce biofilms has been frequently reported in *S. maltophilia* (de Souza et al. 2025) and aligns with the genomic presence of adhesion-related genes, such as *smf-1*, which has been previously associated with biofilm formation and adhesion to both biotic and abiotic surfaces (de Oliveira-Garcia et al. 2003).

A limitation of this study is that the isolation strategy relied on meropenem-selective media, which may have preferentially enriched *Stenotrophomonas* populations with reduced susceptibility or resistance to carbapenems. Consequently, the species composition, sequence type diversity, and antimicrobial resistance profiles described herein should be interpreted within the context of this selective approach and may not be representative of the broader *Stenotrophomonas* population present in the veterinary hospital environment.

Overall, this study expands current knowledge on the occurrence of *Stenotrophomonas* spp. in veterinary hospital-associated environments, which remain poorly explored. The observed intrinsic resistance traits and resistance to multiple antimicrobial classes, combined with the ability to adapt to different environmental niches, highlight the potential of these environmental strains to persist and disseminate antimicrobial and antiseptic resistance determinants.

In this context, our findings provide evidence for the occurrence of environmental *Stenotrophomonas* spp., including *S. maltophilia*, *S. hibiscicola*, *S. forensis*, and *S. muris*, in veterinary hospital facilities, with high sequence type variability. These strains exhibited resistance to clinically important antimicrobials, along with adaptive environmental traits that may contribute to their persistence in hospital environments and facilitate the transmission of antimicrobial resistance determinants to other bacteria. Together, these findings suggest that hospital-associated environments may act not only as reservoirs of resistant *Stenotrophomonas* strains but also as selective niches that contribute to the maintenance and dissemination of antimicrobial resistance among bacterial communities, with potential implications for both human and animal health.

## Supplementary Information

Below is the link to the electronic supplementary material.

Supplementary Material 1 (DOCX 1.21 MB)

## Data Availability

The data underlying this article are available in the National Center for Biotechnology Information under the following accession numbers: JBSURZ000000000, JBSURY000000000, JBWXRT000000000, JBWXRS000000000, JBSURX000000000, JBSURW000000000, JBWXRR000000000, JBSURV000000000, JBSURU000000000, JBWXRQ000000000, JBSURT000000000, JBSURS000000000, JBWXRP000000000, JBWXRO000000000, JBSURR000000000, JBSURQ000000000, and JBWXRN000000000.
