# Supplementary material for: Occurrence of Stenotrophomonas carrying β-lactamase-encoding genes in a veterinary hospital environment
Source: Vet Res Commun. 2026 Sep 23;50(6):588. doi: 10.1007/s11259-026-11539-z (PMC13601196; doi:10.1007/s11259-026-11539-z)
Supplement: Supplementary file 1 [file 11259_2026_11539_MOESM1_ESM.docx]

**Appendix A Supplementary data**

**Occurrence of *Stenotrophomonas* carrying β-lactamase-encoding genes in a veterinary hospital environment**

Maria Eduarda Rocha Jacques da Silva^a,b^, Rafaela da Rosa Marques^a^, Tainara Soares Weyh^a,b^, Júlia Mendes Stropper^a^, Laura Cadó Nemitz^a,b^, Gabriela Merker Breyer^a^, Lívia Kmetzsch^c^, Silvia Dias de Oliveira^d^, Franciele Maboni Siqueira^a,b*^

^a^Veterinary Bacteriology Laboratory, Faculty of Veterinary Medicine, Federal University of Rio Grande do Sul, Porto Alegre 91540-000, Brazil

^b^ Postgraduate Program in Veterinary Science, Federal University of Rio Grande do Sul, Porto Alegre 91540-000, Brazil

^c^Laboratory of Molecular Biology of Pathogens, Center of Biotechnology, Federal University of Rio Grande do Sul, Porto Alegre 91501-970, Brazil

^d^Laboratory of Immunology and Microbiology, School of Health and Life Sciences, Pontifical Catholic University of Rio Grande do Sul, Porto Alegre 90619-900, Brazil

*Corresponding author

E-mail addresses: franciele.siqueira@ufrgs.br

This supplementary material portion contains 3 tables and 1 figures.

| **Table S1** | Antimicrobial susceptibility classification of the strains determined by disk diffusion and MIC. |
| --- | --- |
| **Table S2** | Genome assembly statistics of *Stenotrophomonas* spp. strains |
| **Table S3** | Antimicrobial susceptibility profiles of individual *Stenotrophomonas* strains determined by disk diffusion and MICs of meropenem |
| **Fig. S1** | Curli and cellulose production by Stenotrophomonas spp. strains at different temperatures. (a) Curli production assessed on LB-CRBB agar plates at 28 °C and 37 °C. (b) Cellulose production evaluated on Calcofluor-containing medium at 28 °C and 37 °C. |

| **Table S1**. Antimicrobial susceptibility classification of the strains determined by disk diffusion and MIC. | | | | | | |
| --- | --- | --- | --- | --- | --- | --- |
| **Antimicrobial** | **S** | **I** | **R** | **Interpretive criteria source** | **Target organism** | **Disk content (µg)** |
|  | **(≥ mm)** | **(mm)** | **(≤ mm)** |  |  |  |
| Sulfamethoxazole/trimethoprim | ≥16 | 11-15 | ≤10 | M100 | *Stenotrophomonas maltophilia* | 25 |
| Cefoxitin | ≥18 | 15-17 | ≤14 | M100 | *Enterobacterales* | 30 |
| Ceftazidime | ≥21 | 18-20 | ≤17 | M100 | *Pseudomonas aeruginosa* | 30 |
| Levofloxacin | ≥17 | 14-16 | ≤13 | M100 | *Stenotrophomonas maltophilia* | 5 |
| Ciprofloxacin | ≥25 | 19-24 | ≤18 | M100 | *Pseudomonas aeruginosa* | 5 |
| Gentamicin | ≥16 | 13-15 | ≤12 | VET01S ¹ | *Pseudomonas aeruginosa* | 10 |
| Tobramycin | ≥19 | 13-18 | ≤12 | VET01S ² | *Pseudomonas aeruginosa* | 10 |
| Amikacin | ≥17 | 15-16 | ≤14 | VET01S ² | *Pseudomonas aeruginosa* | 30 |
| Chloramphenicol | ≥18 | 13-17 | ≤12 | M100 | *Enterobacterales* | 30 |
| Doxycycline | ≥14 | 11-13 | ≤10 | M100 | *Enterobacterales* | 30 |
| Tetracycline | ≥15 | 12-14 | ≤11 | M100 | *Enterobacterales* | 30 |
| Ampicillin | ≥17 | 14-16 | ≤13 | M100 | *Enterobacterales* | 10 |
| Amoxicillin/clavulanate | ≥18 | 14-17 | ≤13 | M100 | *Enterobacterales* | 20/10 |
| M100: CLSI M100-ED35:2025 (humans); | |  |  |  |  |  |
| ¹ VET01S: CLSI VET01S-ED7:2024 (dogs); | |  |  |  |  |  |
| ² VET01S: CLSI VET01S-ED7:2024. (humans). | |  |  |  |  |  |

| **Table S2.** Genome assembly statistics of *Stenotrophomonas* spp. strains | | | | | | | | | | | | |
| --- | --- | --- | --- | --- | --- | --- | --- | --- | --- | --- | --- | --- |
| Strain ID | Isolation source | Bacterial species | ST | Contigs | N50 | Total Size (bp) | Coverage (X) | CDS | tRNAs | rRNAs | tmRNA | Genome Acession number |
| D1 | DHU | *S. maltophilia* | 1403 | 68 | 119,56 | 4,489,790 | 382 | 4027 | 67 | 4 | 1 | JBSURZ000000000 |
| D2 | DHU | *S. muris* | 930 | 65 | 172,83 | 4,764,958 | 337 | 4325 | 69 | 3 | 1 | JBSURY000000000 |
| D3 | DHU | *S. muris* | 300 | 52 | 144,26 | 4,830,848 | 332 | 4379 | 66 | 3 | 1 | JBWXRT000000000 |
| D4 | DHU | *S. maltophilia* | 950 | 84 | 139,03 | 4,678,527 | 311 | 4264 | 64 | 4 | 1 | JBWXRS000000000 |
| D5 | DHU | *S. maltophilia* | 1407 | 64 | 134,66 | 4,500,086 | 398 | 4031 | 66 | 4 | 1 | JBSURX000000000 |
| D6 | DHU | *S. maltophilia* | 1407 | 72 | 101,9 | 4,499,917 | 298 | 4027 | 65 | 4 | 1 | JBSURW000000000 |
| D7 | DHU | *S. hibiscicola* | 1402 | 57 | 137,97 | 4,385,522 | 321 | 3956 | 73 | 6 | 1 | JBWXRR000000000 |
| D8 | DHU | *S. maltophilia* | 1399 | 89 | 108,04 | 4,495,796 | 359 | 4004 | 65 | 3 | 1 | JBSURV000000000 |
| D9 | DHU | *S. maltophilia* | 1399 | 75 | 100,04 | 4,490,036 | 362 | 4006 | 68 | 3 | 1 | JBSURU000000000 |
| C1 | CHU | *S. maltophilia* | 1405 | 49 | 142,55 | 4,379,687 | 360 | 3924 | 73 | 4 | 1 | JBWXRQ000000000 |
| W1 | HW | *S. maltophilia* | 212 | 54 | 170,67 | 4,560,940 | 424 | 4145 | 69 | 3 | 1 | JBSURT000000000 |
| W2 | HW | *S. maltophilia* | 212 | 45 | 170,77 | 4,561,363 | 438 | 4149 | 73 | 3 | 1 | JBSURS000000000 |
| W3 | HW | *S. forensis* | 1404 | 48 | 166,03 | 4,868,062 | 312 | 4362 | 66 | 4 | 1 | JBWXRP000000000 |
| W4 | HW | *S. forensis* | 1404 | 47 | 256,32 | 4,871,521 | 343 | 4371 | 65 | 4 | 1 | JBWXRO000000000 |
| W5 | HW | *S. maltophilia* | 770 | 75 | 123,51 | 4,687,671 | 398 | 4227 | 67 | 4 | 1 | JBSURR000000000 |
| W6 | HW | *S. maltophilia* | 1400 | 65 | 104,000 | 4,450,431 | 414 | 3994 | 68 | 4 | 1 | JBSURQ000000000 |
| W7 | HW | *S. hibiscicola* | 1402 | 51 | 157,900 | 4,314,025 | 382 | 3866 | 59 | 4 | 1 | JBWXRN000000000 |
| DHU = dog hospitalization unit; CHU = cat hospitalization unit; HW = hospital wastewater; ST = sequence typing | | | | | | | | | | | |  |

| **Table S3**. Antimicrobial susceptibility profiles of individual *Stenotrophomonas* strains determined by disk diffusion and MICs of meropenem | | | | | | | | | | | | | | | | |
| --- | --- | --- | --- | --- | --- | --- | --- | --- | --- | --- | --- | --- | --- | --- | --- | --- |
| Strain ID | Bacterial species | Isolation source | Disk diffusion | | | | | | | | | | | | | MIC |
|  |  |  | Aminoglycosides | | | Cephalosporins | | Amphenicols | Fluoroquinolones | | Sulfonamides | Tetracyclines | | Penicillins | β-lactamase inhibitor combinations | Carbapenems |
|  |  |  | GEN | TOB | AMK | CFO | CAZ | C | LVX | CIP | TMP-SMX | DOX | TET | AMP | AMC | MER |
| D1 | *S. maltophilia* | DHU |  |  |  |  |  |  |  |  |  |  |  |  |  | 128 µg/mL |
| D2 | *S. muris* | DHU |  |  |  |  |  |  |  |  |  |  |  |  |  | 128 µg/mL |
| D3 | *S. muris* | DHU |  |  |  |  |  |  |  |  |  |  |  |  |  | 32 µg/mL |
| D4 | *S. maltophilia* | DHU |  |  |  |  |  |  |  |  |  |  |  |  |  | 128 µg/mL |
| D5 | *S. maltophilia* | DHU |  |  |  |  |  |  |  |  |  |  |  |  |  | 64 µg/mL |
| D6 | *S. maltophilia* | DHU |  |  |  |  |  |  |  |  |  |  |  |  |  | 64 µg/mL |
| D7 | *S. hibiscicola* | DHU |  |  |  |  |  |  |  |  |  |  |  |  |  | >128 µg/mL |
| D8 | *S. maltophilia* | DHU |  |  |  |  |  |  |  |  |  |  |  |  |  | 128 µg/mL |
| D9 | *S. maltophilia* | DHU |  |  |  |  |  |  |  |  |  |  |  |  |  | 64 µg/mL |
| C1 | *S. maltophilia* | CHU |  |  |  |  |  |  |  |  |  |  |  |  |  | 128 µg/mL |
| W1 | *S. maltophilia* | HW |  |  |  |  |  |  |  |  |  |  |  |  |  | 128 µg/mL |
| W2 | *S. maltophilia* | HW |  |  |  |  |  |  |  |  |  |  |  |  |  | 128 µg/mL |
| W3 | *S. forensis* | HW |  |  |  |  |  |  |  |  |  |  |  |  |  | 128 µg/mL |
| W4 | *S. forensis* | HW |  |  |  |  |  |  |  |  |  |  |  |  |  | 128 µg/mL |
| W5 | *S. maltophilia* | HW |  |  |  |  |  |  |  |  |  |  |  |  |  | 64 µg/mL |
| W6 | *S. maltophilia* | HW |  |  |  |  |  |  |  |  |  |  |  |  |  | 128 µg/mL |
| W7 | *S. hibiscicola* | HW |  |  |  |  |  |  |  |  |  |  |  |  |  | >128 µg/mL |
| Gray squares: antimicrobial resistance by disk-diffusion assay | | | | | | | | | | | | | | | |  |
| DHU = dog hospitalization unit; CHU = cat hospitalization unit; HW = hospital wastewater; GEN = gentamicin; TOB = tobramycin; AMK = amikacin; CFO = cefoxitin; CAZ = ceftazidime; C = chloramphenicol; LVX = levofloxacin; CIP = ciprofloxacin; TMP-SMX = trimethoprim–sulfamethoxazole; DOX = doxycycline; TET = tetracycline; AMP = ampicillin; AMC = amoxicillin/clavulanate; MER = meropenem | | | | | | | | | | | | | | | | |

**Fig. S1.** Curli-associated and cellulose-like phenotypes of Stenotrophomonas spp. strains at different temperatures. (a) Colony morphotypes on LB-CRBB agar plates at 28 °C and 37 °C, representing curli-associated phenotypes. (b) Calcofluor binding on Calcofluor-containing medium at 28 °C and 37 °C, representing cellulose-like phenotypes.
